# A systematic review and meta-analysis of the prevalence of therapeutic targets in cervical cancer

**DOI:** 10.3332/ecancer.2021.1200

**Published:** 2021-03-09

**Authors:** Maria Guadalupe Patrono, Maria Florencia Calvo, Juan Victor Ariel Franco, Virginia Garrote, Valeria Vietto

**Affiliations:** 1 Department of Gynecology, Hospital Italiano de Buenos Aires, Gascon 450, Buenos Aires C1181ACH, Argentina; 2 Family and Community Medicine Division, Hospital Italiano de Buenos Aires, Gascon 450, Buenos Aires C1181ACH, Argentina; 3 Argentine Cochrane Centre, Instituto Universitario Hospital Italiano, Potosí 4265, Buenos Aires C1199ABB, Argentina; 4 Central Library, Hospital Italiano de Buenos Aires, Instituto Universitario Hospital Italiano de Buenos Aires, Tte. J. D. Perón 4190, 1º floor, stair J. C1199ABB, Argentina; a https://orcid.org/0000-0002-1309-2114; b https://orcid.org/0000-0002-2224-1564; c https://orcid.org/0000-0003-0411-899X; d https://orcid.org/0000-0002-7328-6228; e https://orcid.org/0000-0003-4619-9812

**Keywords:** cervical cancer, cervical neoplasia, mutation, prevalence, therapeutic targets

## Abstract

Cervical Cancer (CC) is a significantly prevalent disease in developing countries. Currently, targeted therapies are not a primary standard of care in CC. This information could be crucial for developing directed therapies and patient screening for biomarkers that would allow personalised treatment of CC. This systematic review aimed to estimate the prevalence of potential therapeutic targets such as the epidermal growth factor receptor (EGFR) and the PI3K/Akt/mTOR and Ras/Raf/MAPK pathways in patients with CC, identified through genomic and non-genomic testing. Studies were identified through an ad-hoc search strategy from the available on MEDLINE (Ovid), CENTRAL, LILACS, SCOPUS, through the Clinical Trial registry on Clinicaltrials.gov, International Clinical Trials Registry Platform, RENIS (Argentine National Registry of Health Research) and grey literature sources. We included 74 studies which represented a total pool of 7,862 participants. Forty-five studies informed mutations of EGFR, with a combined positivity rate of 53% (95%CI: 45%–60%; I^2^ = 95%). Twenty studies informed the presence of mutations in PIK3CA with a combined positivity rate of 30% (95%CI: 21%–39%; I^2^ = 96%). Twenty-three studies reported a mutation in Ras, with a combined positivity rate of 14% (95%CI: 8%–21%; I^2^ = 95%). Raf mutations were informed in six studies. Six studies informed the presence of Akt mutations, two studies informed mTOR mutations and only one study reported mutations of MAPK. The most frequently described therapeutic targets were EGFR, and the PIK3CA and Ras pathways, though inconsistency in positivity rates was significant. Our study did not allow the identification of any specific clinical characteristics that might explain the observed heterogeneity. Despite the overall good quality of the included studies, the applicability of these results to patients’ general population with CC is still unclear.

## Introduction

Cervical-uterine cancer is the second most diagnosed cancer in women in developing countries. About 4,400 new cases are diagnosed in Argentina, and approximately 1,800 women die from this disease each year. Globally, in 2018, 570,000 women were diagnosed with cervical cancer (CC), and 311,000 died from this cause, constituting the fourth in frequency among women and the fourth as a cause of death. Approximately 84% of all CCs and 88% of all deaths caused by this cancer occurred in lower resource countries [1–3].

Infection by human papillomavirus (HPV) has been clearly established as a necessary cofactor for the development of CC. The use of cervical cytology, known as Papanicolaou testing, as well the addition of HPV co-testing has significantly improved the detection and treatment of precursor and preinvasive cervical lesions, allowing to identify patients who are at greater risk of developing CC [1].

Standard treatment for women with early-stage CC (International Federation of Gynecology and Obstetrics (FIGO) stages IA and IB) consists of radical hysterectomy or concurrent radiotherapy/chemotherapy treatment. Survival rates are similar with either treatment modality. The choice of therapy usually depends on either treatment’s adverse event profile, the presence or absence of specific prognostic factors, patient or physician preference and access to the different therapeutic options [2]. In patients presenting with advanced stages of CC, both neoadjuvant chemotherapy and concurrent radiotherapy/chemotherapy are suitable alternatives. Still, neoadjuvant chemotherapy before surgery or chemoradiation is associated with inferior outcomes compared to concurrent chemoradiation [2, 4, 5].

Nonetheless, therapeutic options have been evolving continuously, along with the discovery and development of targeted therapies. Genetic profiling of tumours could predict sensitivity or resistance to these novel treatments, facilitating a personalised approach for each patient. Identifying specific genetic tumour alterations or the presence of distinct targets can guide treatment with molecular agents and provide prognostic information about the disease [2, 6, 7].

In 1997, the Food and Drug Administration of the United States of America approved the first targeted therapy, rituximab (Rituxan®), to treat patients with Non-Hodgkin B-cell lymphoma who had progressed to other treatments. Currently, numerous tumours such as breast, ovarian, liver, prostate, lung cancer and melanoma have targeted therapies, which have proven beneficial in improving disease-free survival and overall survival in some cases. Regarding CC, one of the first studies investigating the use of the specific anti-PDLI-1 monoclonal antibody Pembrolizumab in patients with recurrent disease was published in 2019 [8].

However, targeted therapies have not yet been established as a primary treatment standard in CC. One of the main limitations for the development of directed therapy is that the prevalence of mutations or specific molecular targets in CC is still unclear, thus making it impossible to screen and identify patients who may be suitable candidates. The characterisation of the most frequent mutations in patients with CC is necessary. It will allow the development of detection kits for specific markers with prognostic and predictive potential, which may be used in clinical practice to guide and personalise oncologic treatment for each patient.

Well-established markers as therapeutic options in many tumours include the epidermal growth factor receptor (EGFR, ErbB), phosphatidylinositol 3-kinase (PI3K)/Akt/mTOR and Ras/Raf/MAPK [9–11]. The epidermal growth factor receptor (EGFR, ErbB) belongs to the superfamily of plasma membrane-localised receptors with intrinsic tyrosine kinase activity, activated by the epidermal growth factor (EGF) and the tumour growth factor alpha. EGFR is activated by dimerisation, which depends on ligand binding, although it can also occur when there is overexpression and structural alterations of the receptor. Signalling through the EGFR is crucial in embryonic development: in epithelial development, cell proliferation and organogenesis. Uncontrolled EGFR activity (by overexpression or structural abnormalities in the receptor or its ligands) has been implicated in many aspects of tumour growth, including the promotion of cell proliferation, angiogenesis, invasion, metastasis and survival. When an extracellular ligand binds to EGFR, activation of its tyrosine kinase and phosphorylation of tyrosine residues occur. The best known and best-characterised signalling pathway initiated by activated EGFR is the Ras/MAPK pathway, which appears to be essential for EGF-mediated cell proliferation. Another important pathway is the PI3K, and the PI3K/Akt/mTOR cascade, which results in increased protein transcription, synthesis and proliferation. The PIK3CA gene encodes the p110 alpha catalytic subunit of PI3K, which is often mutated in tumoural cells [12–15].

This systematic review aimed to estimate the prevalence of potential and established therapeutic targets in patients with CC. We considered the following markers: EGFR, and both PI3K/Akt/mTOR and Ras/Raf/MAPK intracellular pathways.

## Materials and methods

A systematic review was performed, according to the methodologic guidelines of the Manual for Systematic Reviews of Observational Studies of the Joanna Briggs Institute [16, 17] and the checklist for Preferred Reporting Items for Systematic Reviews and Meta-analyses (PRISMA) [18]. The study protocol was approved by the Internal Review Board of the Hospital Italiano de Buenos Aires (Ethics Committee in Research Protocols, CEPI) and was registered in the PROSPERO database of systematic reviews (ID CRD42017067538).

### Search methods

The search was performed using the following electronic databases: MEDLINE (Ovid), CENTRAL, LILACS, SCOPUS and the registry of Clinical Trials Clinicaltrials.gov, the International Clinical Trials Registry Platform (ICTRP) and RENIS until September 2019. The search strategy was designed and executed by a librarian specialised in systematic reviewing. The full search strategy, which was initially developed for MEDLINE (Ovid) and later adjusted for the other search engines, is listed in Appendix A. Searches were also performed in grey literature sources, through Google Scholar and OpenGrey. The references of the included studies were revised, and the summaries and abstracts of the most relevant speciality meetings and conferences of the past 5 years were manually explored: Annual Meeting of the American Society of Gynecologic Oncologist, Annual Meeting of the International Gynecologic Cancer Society, Annual Meeting of European Society of Medical Oncology and Annual Meeting of the American Society of Clinical Oncology.

### Study selection criteria

For this systematic review, the inclusion of primary studies was considered for randomised and quasi-randomised controlled trials, observational cohort studies and cross-sectional studies, including patients of any age, diagnosed with primary, non-recurrent squamous, adenosquamous or adenocarcinoma CC, in whom expression of one or more therapeutic or potential therapeutic targets were explored. Surface protein expression and intracellular genomic expression and amplification were both considered positive results. The investigated targets were the following: EGFR and both PI3K/Akt/mTOR and Ras/Raf/MAPK pathways, with or without HPV virus typification. Studies performed in commercial cell cultures, patients with CC recurrence and those with unavailable or incomplete data to estimate the proportion of participants with a positive test were excluded. Inclusion was limited to English or Spanish reports.

### Study selection procedure

Study screening and selection were performed by two independent reviewers (MGP and MFC), using the online platform COVIDENCE. The first stage involved evaluating the titles and abstracts of all records retrieved by the search strategy. The second stage consisted of the full-text revision. The full texts considered relevant after initial screening were recovered and analysed according to the research question. Reviewer discrepancies were resolved by consensus or by a third reviewer (VV).

### Data extraction

Data extraction was performed by completing an *ad-hoc* standardised electronic form (Google Form®), after independent proofing by two reviewers (MGP and MFC). Discrepancies were resolved by consensus or by a third reviewer (VV).

The following data were collected: study design and aim, language, year of publication, inclusion and exclusion criteria and characteristics of the study population, which included sample size, age at diagnosis, tumour histology (squamous, adenosquamous or adenocarcinoma) stage of disease at diagnosis according to the International Federation of Gynecology and Obstetrics staging criteria and type of treatment. Additional variables that were documented were tumour size, parametrial invasion, depth of myocervical invasion, lymphovascular space invasion, presence of pelvic or paraaortic lymph node metastases, adjuvant treatment and HPV typification.

The primary outcome was to describe the prevalence of targets or potential targets, defined as positive expression (either genomic or nongenomic) of one or more of the following markers, independent of histologic tumour subtype or stage: EGFR and both PI3K/Akt/mTOR and Ras/Raf/MAPK pathways.

### Strategies for data synthesis

Statistical analysis was performed using STATA® software (StataCorp, College Station, TX). The total percentage of variation among studies due to heterogeneity was assessed using the I² test. Low heterogeneity was defined for values between 0% and 40%, moderate for 30% through 60%, substantial between 50% and 90% and considerable heterogeneity was established between 75% and 100%. Since high heterogeneity was expected, proportions were combined through meta-analysis using a random-effects model [19]. Combined estimations were calculated with 95% confidence intervals and were represented through forest plot. We attempted to explore heterogeneity by performing prespecified subgroup analysis, for both histologic subtype and detection method (genomic versus nongenomic). However, the data were insufficient for performing these analyses or did not gather enough power to detect differences.

### Sensibility analysis

Performance of sensitivity analysis was intended at the study onset, in order to explore the risk of possible selection and information bias influencing the proportion of positivity of the detection tests for the therapeutic targets. These analyses were not performed since none of the included studies was considered to be at high risk of bias in either domain.

### Assessment for publication bias

Funnel plots were employed to evaluate small studies’ effect whenever at least ten studies were available for a specific outcome.

### Assessment of study quality and bias

The methodological quality of the included studies was independently assessed by each reviewer, by using the ‘Critical Appraisal by the Joanna Briggs Institute for prevalence studies’ tool, adapted *ad-hoc* for this review [20, 21]. Through this structured and standardised instrument consisting of seven specific questions, key aspects of the study population, setting and design were explored, such as detailed population description, adequate, appropriate and representative sampling, standardised measurement of outcomes and statistical aspects, including appropriate sample size and accurate use of statistical methods for data extraction (Appendix C).

## Results

### Study selection and inclusion

The study selection process is summarised in Figure 1. After executing the search strategy, which is described in Appendix A, a total of 3,935 studies were retrieved. Among these, 1,256 were automatically detected as duplicates by the reference management software and excluded. Manual search did not find additional studies.

After the initial title and abstract screening process of all the initially recovered articles, 167 studies were selected for full-text review. After this stage, 74 studies that gathered the established inclusion criteria for our revision were included. The complete list of the 93 excluded studies can be found in Appendix B. The studies are grouped according to the reason for exclusion, which included impossibility of access to the full text (in all cases, direct email contact with the author was attempted but not achieved), inappropriate study design (narrative or systematic reviews), inappropriate study population (e.g. mutations described in cell cultures or cell lines or tumour histologies other than the prespecified types) and those that did not allow confidable data extraction.

### Characteristics of the included studies

The 74 included (Table 1) studies were published between 1989 and 2019. Thirty-nine of the studies had a cross-sectional design (52.7%), 33 (44.6%) were observational cohort studies and 2 (2.7%) were randomised clinical trials, which resulted in a pooled total of 7,862 patients (median: 106, interquartile range: 30–110, range: 7–854). Forty-five studies (60.8%) specified patient age, with a resulting mean of 49 years (standard deviation: 5.73, range: 39–69).

With regard to the primary outcome, 57 studies (77%) assessed the prevalence of or proportion of specific mutations, 5 (6.7%) evaluated response to treatment, 8 (10.8%) focused on patient outcome and the remaining 4 had other non-clinically applicable aims (5.4%).

English was the preferred language for publication in 71 articles (96%). The remaining 3 were in Spanish. Only 4 studies (5.4%) were multicentric, led by investigators from different countries, while 70 were performed in single countries (94.6%). The study population was grossly represented by patients with CC (64 studies; 86.4%), but four studies (5.4%) also included patients with precursor lesions and five (6.7%) involved patients with other gynaecologic cancers (including breast), and one study included other solid tumours (pancreas and colon, among others).

Tumour histology data was not extractable in seven studies (9.4%), representing 752 patients. The remaining studies pooled a total of 6,886 (87.6%) patients, among which 5,377 (68.4%) had squamous histology, 394 (5%) had adenosquamous subtypes and 1,115 (14.2%) were adenocarcinomas.

Thirty-seven studies (50%) detailed the type of treatment received by the study population, whether it was concomitant chemo/radiation therapy, neoadjuvant chemotherapy or primary surgery. Forty-two studies (56.7%) included data regarding patient outcome and prognosis, such as disease-free survival or overall survival. Twenty-two studies (29.7%) also stated the study population’s HPV status and several specified subtypes.

In six of the included studies (8.1%), the authors declared conflicts of interest. Twenty-six declared having no disclosures whatsoever (35.1%). In the remaining studies, this information was unavailable. Regarding funding and grant support, 10.2% received public funding, 10.2% received private grants and 71.6% had a combined source, through grants from universities, laboratories or scholarships. Funding was unspecified in five studies (9.5%).

### Methodological quality of the included studies

In all of the included studies, the sample population was found to adequately represent the target population (criteria 1), which means that the patients with CC included, complied with the selected subtypes’ prerequisite (squamous, adenosquamous or adenocarcinoma). The majority of the studies (97%) recruited the study population adequately (criteria 2), so the risk of selection bias was considered low.

Less than 6% of the included studies reported accurate sample size calculation (criteria 3), while in most cases (94%) this parameter was not assessable, given that the method for the definition of sample size was either not informed or was not considered applicable to the primary aim of the study.

The majority of the studies (77%) accurately described the study population (criteria 4). It was partially described in 13.5% and 9.5% did not provide any information regarding the characteristics of the population. All of the included studies (100%) performed data analysis with adequate coverage of the population and used one or more of the prespecified standard methods for measuring the variable of interest (100%), which is why the risks of data loss and information bias were considered low (criteria 5 and 6).

In the vast majority of the included studies, the described statistical analysis method was adequate for the study aim (93%). Statistical analysis was unclear or uninformed in 5.4% and in one case, was considered inadequate by both reviewers (criteria 7) (Figure 2).

### Revision findings

The following is a description of the results for each specific therapeutic or potential therapeutic target.

#### EGFR

Forty-five studies (61%) representing a combined population of 3,605 patients informed the presence of this mutation [6, 22, 23, 25, 26, 29, 34, 35, 38–42, 44–49, 51, 53–56, 58–62, 65–69, 71, 74, 77, 78, 80–83, 88, 90, 92]. The combined positivity rate was 53% (95%CI: 45%–60%; I^2^ = 95%; Figure 1, Appendix D). Nonetheless, considering the substantial heterogeneity, the positivity rate varied from 4% to 100%. Potential explanations for this heterogeneity were explored, but no differences relative to histological subtype, testing method (genomic versus non-genomic) or any other clinical characteristics were established (Figure 2, Appendix D).

#### PIK3CA

Twenty studies (27%) pooling 2,993 participants informed the presence of this mutation [15, 24, 27–33, 35–37, 50, 52, 57, 64, 79, 85, 87, 89]. The combined positivity rate was 30% (95%CI: 21%–39%; I^2^ = 96%; Figure 4, Appendix D). Considering the substantial heterogeneity, the positivity rate varied between 6% and 81%. Possible explanations for this heterogeneity were explored, but no differences for histologic subtypes were found (Figure 5, Appendix D). The influence of the type of test used was not tested due to the insufficient number of studies in each subgroup, nor the observed inconsistency could be attributed to any other clinical condition.

#### Ras

Twenty-three studies (31%) with 2,983 participants reported this mutation [6, 13, 15, 30, 31, 35, 37, 42, 43, 62, 63, 70, 72, 73, 75–77, 79, 84, 86, 87, 89, 91]. The combined positivity rate was 14% (95%CI: 8%–21%; I^2^ = 95%, Figure 7, Appendix D); however, considering the substantial, heterogeneity, the positivity rate ranged between 0% and 82%. When possible explanations for this heterogeneity were explored, higher positivity rates for this marker were found in the squamous subtype than in adenocarcinomas (pooled estimates 6% versus 30%, *p* < 0.01; Figure 8, Appendix D). Also, studies that used non-genomic testing described higher positivity rates than those that employed genomic tests (pooled estimates 13% versus 39%, *p* < 0.01; Figure 9, Appendix D). No additional clinical characteristics that could account for the observed heterogeneity were identified.

#### Akt

Six studies (8%) gathering 1,106 participants informed the presence of this mutation [24, 27, 29, 46, 48, 57]. A quantitative synthesis of all studies was not performed since two different groups were clearly identified; four studies with 970 participants informed very low positivity rates (globally 2%; 95%CI: 0%–5%) and two studies including 136 participants reported high positivity rates (total 88%; 95%CI: 82%–93%) (Figure 11, Appendix D). No clinical characteristics that could explain this inconsistency were identified.

#### Raf

Raf mutations were informed in six studies (8%) with a total of 349 participants [6, 15, 27, 37, 42, 79]. The pooled estimate for positivity rate was 1% (95%CI: 0%–4%; I^2^ = 38%; Figure 12, Appendix D). No clinical characteristics that could explain this inconsistency were identified.

#### mTOR

Two studies (2.7%) reported this mutation with different results [6, 29]. Muller *et al* [6] informed that the positivity rate was 3% (1/29 study subjects), while Bumrungthai *et al* [29] reported a 61% rate (64/105 subjects). No clinical characteristics that could explain this inconsistency were identified.

#### MAPK

Only one study with 105 participants (1.3%) [29] reported this mutation with a positivity rate of 68% (*n* = 71).

### Risk of publication bias

**Figure 3.**
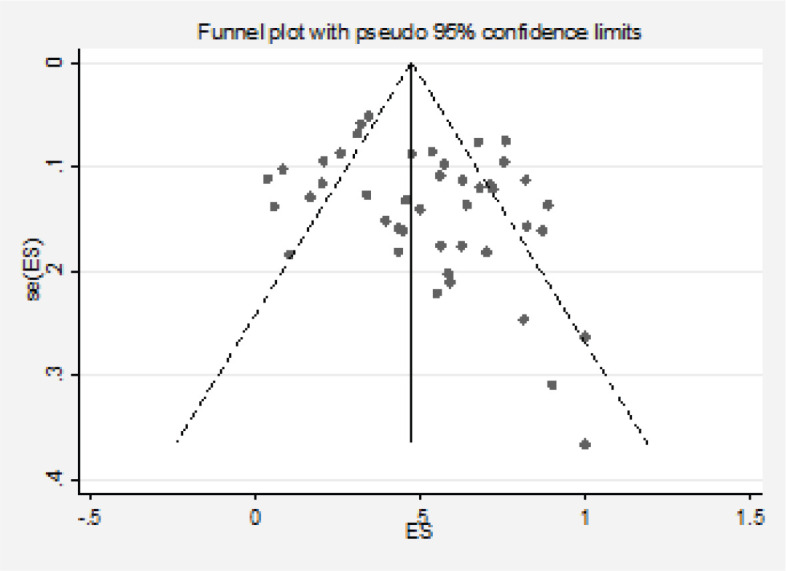
Analysis of the effect of small studies on the EGFR positivity rate (assessment of publication bias).

**Figure 4.**
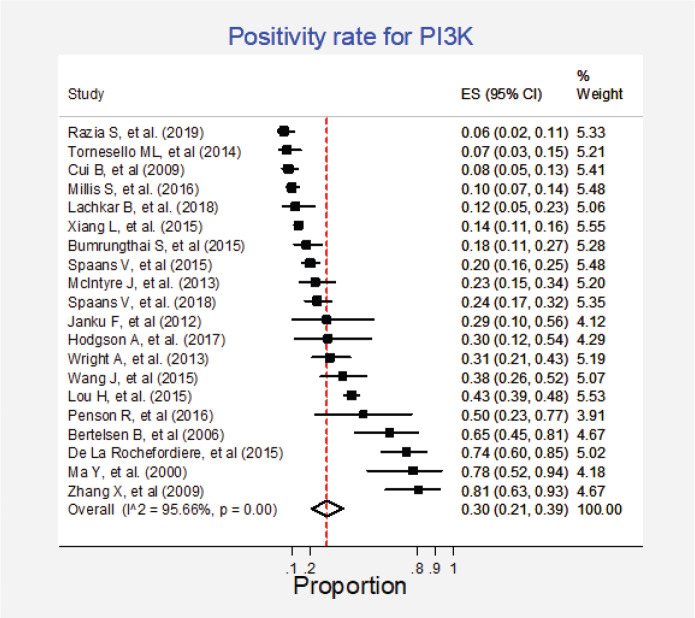
Meta-analysis of the PI3K positivity ratios.

**Figure 5.**
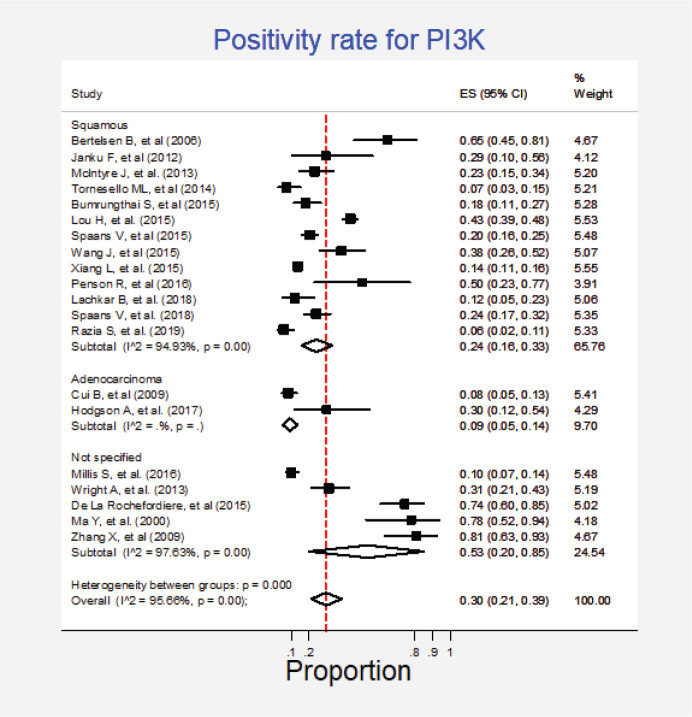
Subgroup analysis. Positivity for PI3K according to the type of histology.

**Figure 6.**
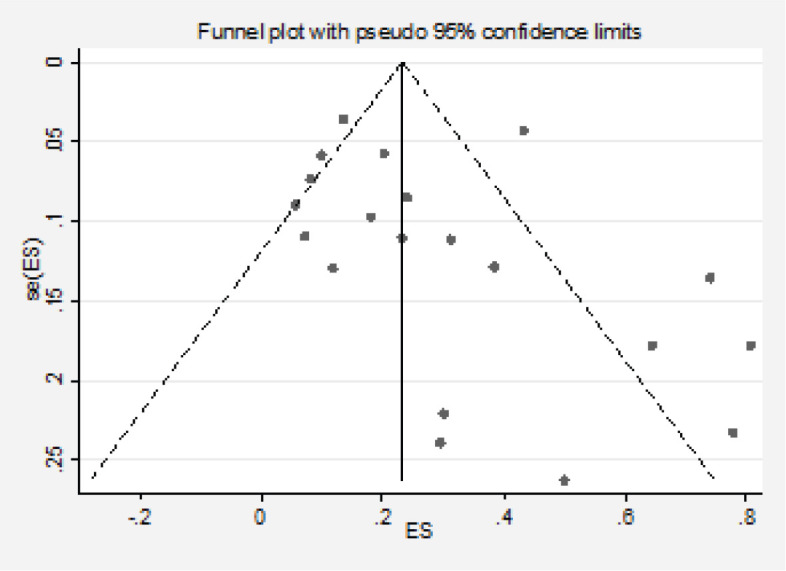
Analysis of the effect of small studies on the PI3K positivity rate (assessment of publication bias).

**Figure 7.**
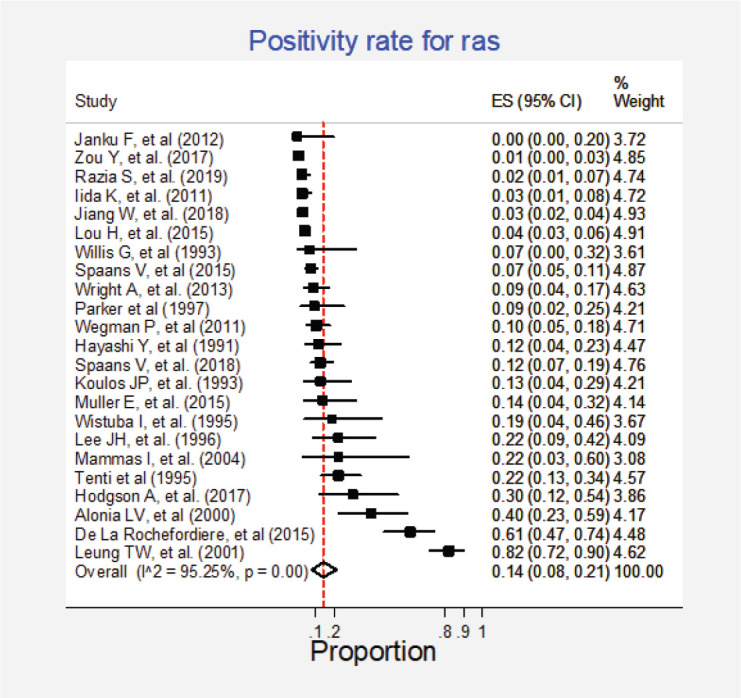
Meta-analysis of the Ras positivity proportions.

**Figure 8.**
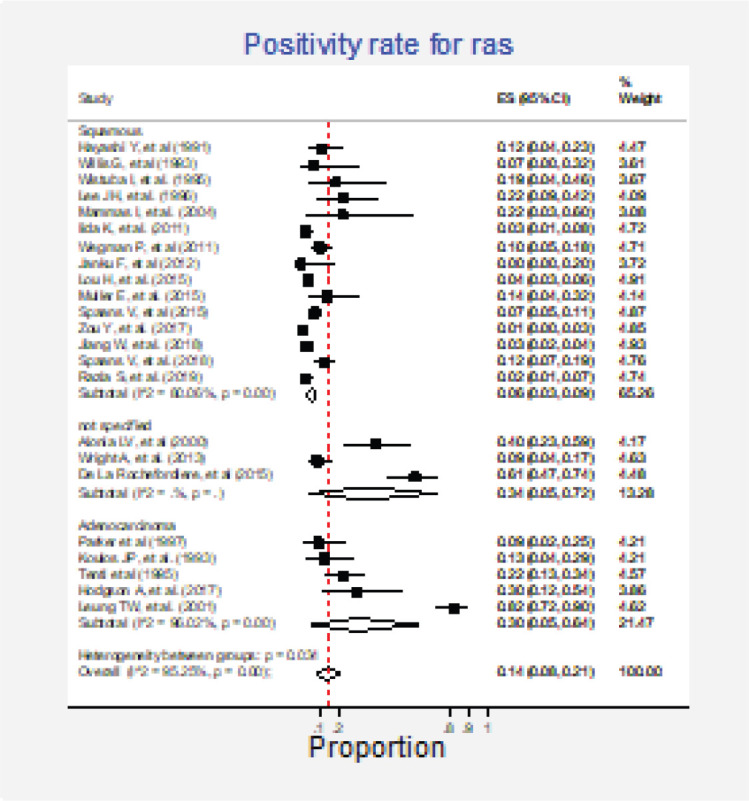
Subgroup analysis. Positivity for Ras according to the histological type.

**Figure 9.**
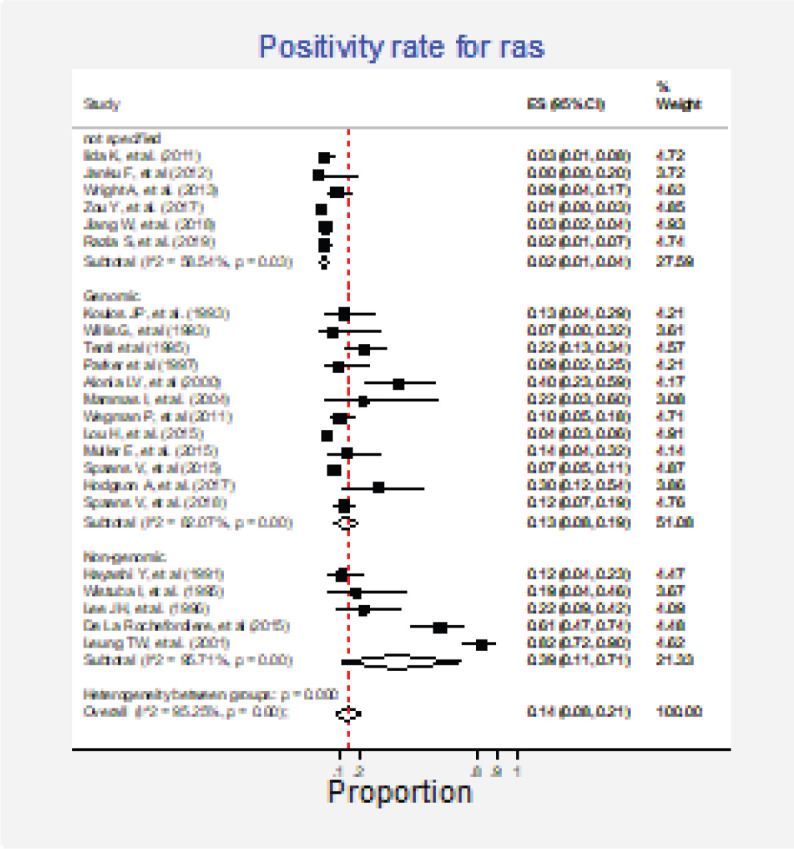
Subgroup analysis. Positivity for Ras according to the type of detection test (genomic versus non-genomic).

**Figure 10.**
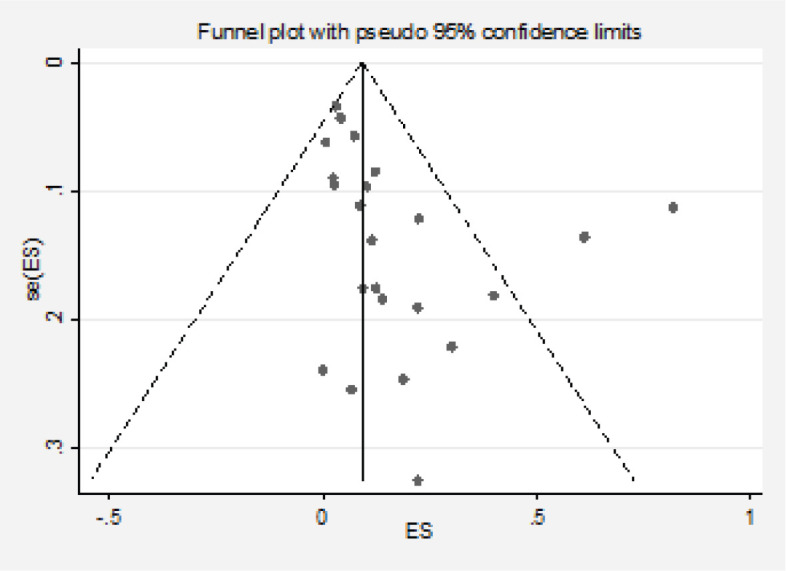
Analysis of the effect of small studies on the Ras positivity rate (assessment of publication bias).

**Figure 11.**
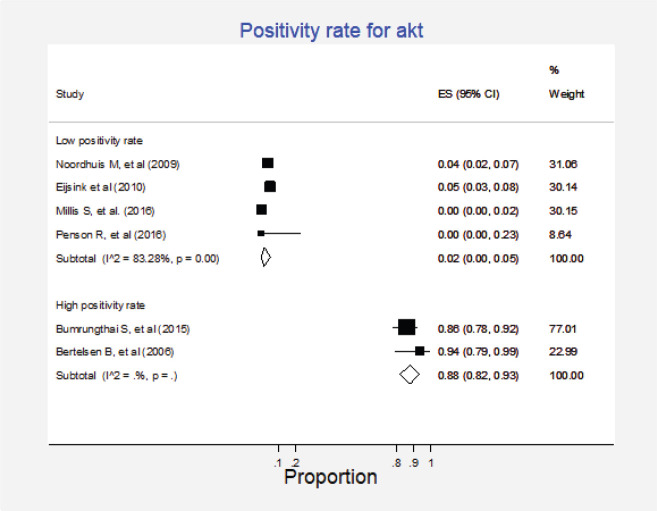
Meta-analysis of the positivity proportions of Akt.

**Figure 12.**
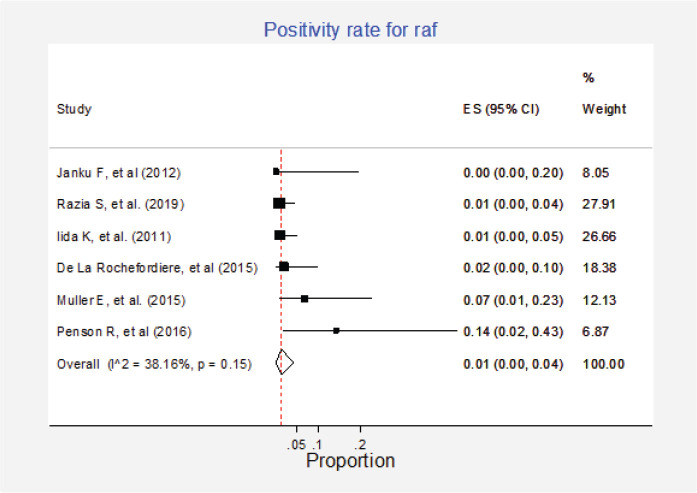
Meta-analysis of the Raf positivity ratios.

We detected significant asymmetry in the funnel plots, which could indicate the presence of publication bias for the mutation positivity proportions of EGFR and PIK3CA, while the asymmetry was less evident for Ras (Figures 3, 6 and 10, Appendix D). Given that the plots can only be represented in outcomes that group at least ten studies, we could not assess the effect of the smaller studies for the remaining markers.

## Discussion

To the authors’ knowledge, this is the first systematic review evaluating the prevalence or proportion of potential and established therapeutic targets in CC. The identification of these markers may allow the development of future research with crucial clinical applicability, such as the screening for specific predictive factors at the onset of treatment, as is already being performed in other tumours. This may ultimately lead to the improvement of clinical outcomes in patients diagnosed with this disease through the use of precision medicine. One example of the benefit of using target therapy to improve outcomes in a different clinical setting is the higher progression-free survival and quality of life by targeting BRAF mutations in patients with metastatic colon cancer. This practice, which currently constitutes a standard of care, allows the identification of patients who will benefit from monoclonal antibody therapy targeting EGFR (cetuximab, panitumumab, among others) [9].

According to our findings, the most frequently explored markers in patients with CC are the EGFR and both PIK3CA and Ras pathways. We were unable to identify clinical characteristics that might justify the substantial inconsistencies observed among these studies for most markers. However, in the case of Ras, we found that the histologic subtype and the nature of the detection test employed could affect the positivity rate of this target (higher in squamous carcinoma and with the use of non-genomic testing). Because of this, the pooled estimates for the prevalence of therapeutic targets were considered to be low-certainty evidence, and the value ranges extracted from primary studies should be highlighted as the main features of this systematic review.

We considered both surface expression and intracellular gene amplification positive results since research is underway in both tumour genome and surface markers that may prove useful for the identification of clinically significant alterations that will probably become relevant in personalising patient treatment and care.

Among the limitations of our study, we can describe the limitations due to the unavailability of language translation resources, which narrowed our options to including only full texts published in English or Spanish. However, we only excluded eight studies for this reason, which is why we consider that the potential impact of this limitation is probably insignificant.

On the other hand, the methodological quality appraisal and the evaluation of the risk of bias in the collected evidence may be somewhat limited. One of the methodological difficulties of systematic reviews of prevalence resides in the diversity of instruments for the appraisal of primary studies. This is in part because there is a lack of consensus regarding which domains need to be evaluated in this study type, and there is no universally accepted assessment tool. For this review, we decided to create an *ad-hoc* adaptation of the ten criteria of the ‘Joanna Briggs Institute Critical Appraisal Checklist for Prevalence Studies’, since we considered it to be the most applicable to our study aim. This structured assessment tool was developed for any clinical condition based on the systematic review of previous instruments. Its validity, applicability and acceptability have been appreciated by experts and may be currently considered the most complete appraisal tool according to a recent review [93].

In general, the quality of the studies included in this revision was adequate. One of the greatest weaknesses that we identified through this instrument was the lack of sample size estimation, which turned out to be a frequent finding in our study pool. Most of the studies reported results in terms of proportions and had not been designed to determine the prevalence of the evaluated markers within the universe of patients with CC. Also, the primary studies did not apply sampling techniques that could guarantee the representativity of the analysed specimens, allowing the generalisation of their results.

The development of directed therapies requires the identification of adequate markers. These are specific targets or checkpoints that play a crucial role in malignant cell proliferation and growth. The assessment of the prognostic and predictive clinical value of these markers lies without the scope of this systematic review and may be an appropriate subject of further research. However, the use of these treatments in other tumours has proven a significant impact on patient outcomes and quality of life and hopefully, this may be soon applicable to CC patients as well.

From a health economics standpoint, it is important also to consider the costs associated with the systematic detection of the targets to implement precision therapies. This will require the performance of economic assessments and the development of clinical trials with patient-centred outcomes that will assess the financial burden of the specific interventions that may result beneficial in each particular context.

## Conclusion

This systematic review found that the most frequently described therapeutic targets were EGFR and the PIK3CA and Ras pathways. Nonetheless, the reported estimates of positivity prevalence for these markers are inconsistent. Our study did not allow the identification of any specific clinical characteristic that might explain the observed heterogeneity, except for the Ras pathways, which showed higher rates of positivity in squamous histologic subtypes and when non-genomic assays were used for testing. Despite the overall high quality of the included studies, the applicability of these results to patients’ general population with CC is still unclear.

## Authors’ contributions

Concept and design: Patrono MG, Calvo MF, Franco JV, Vietto V.

Acquisition, analysis or interpretation of data: Patrono MG, Calvo MF, Franco JV, Garrote V, Vietto V.

Drafting of the manuscript: Patrono MG, Calvo MF, Vietto V.

Critical revision of the manuscript for important intellectual content: Patrono MG, Calvo MF, Franco JV, Garrote V, Vietto V.

Statistical analysis: Franco JV, Vietto V.

Supervision: Vietto V.

## Conflicts of interest

The authors have no conflicts of interest to declare.

## Funding statement

None.

## Figures and Tables

**Figure 1.**
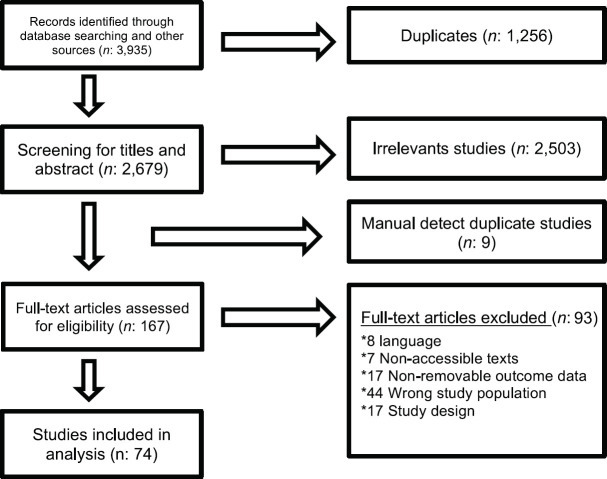
PRISMA flow diagram.

**Figure 2.**
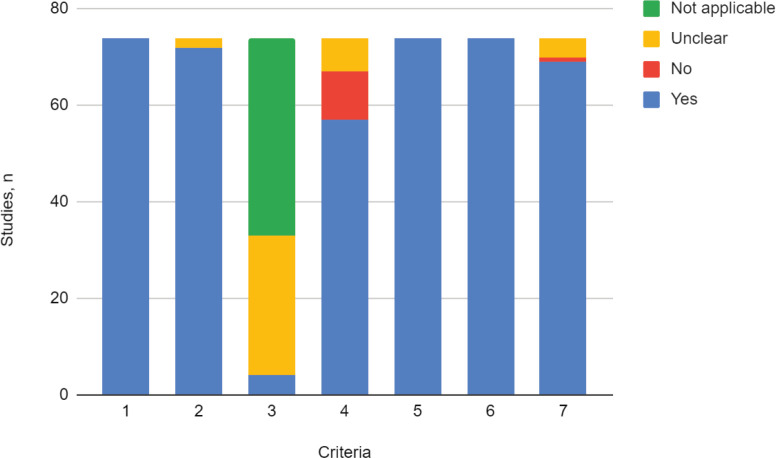
Critical appraisal. Adaptation of the criteria of the ‘Joanna Briggs Institute Critical Appraisal Checklist for Prevalence Studies’. 1. Was the sample frame appropriate to address the target population? 2. Were study participants recruited in an appropriate way? 3. Was the sample size adequate? 4. Were the study subjects and setting described in detail? 5. Was data analysis conducted with sufficient coverage of the identified sample? 6. Was the condition measured in a standard, reliable way for the variable of interest in all cases? 7. Was there appropriate statistical analysis?

**Table 1.** Characteristics of the included studies.

Studies/year	Study design	Objective	Inclusion criteria	*n*	Therapeutics target/potentials therapeutic target	Financial found	Countries
Noh *et al* [22]	CS	RT	CC	20	EGFR	Pri	Korea
Kim *et al* [23]	CSe	Other	CC	16	EGFR	M	Korea
Millis *et al* [24]	CSe	Pr	Solids tumours	291	PI3K Akt	M	>60 countries
Qureshi *et al* [25]	CSe	Pr	CC	95	EGFR	M	India
Hernowo *et al* [26]	CSe	Pr	CC	32	EGFR	NI	Indonesia
Penson *et al* [27]	CSe	Pr	CC, EC, OC, BC	14	PI3K Raf	M	USA
Xiang *et al* [28]	CS	Pr	CC	771	PI3K	Pri	China
Bumrungthai *et al* [29]	CSe	Pr	CC	105	EGFR PI3K Akt mTOR MAPK	Pri	Thailand India USA
Spaans *et al* [30]	CS	Pr	CC	301	PI3K Ras	M	Netherlands
Muller *et al* [6]	CSe	Pr	CC	29	EGFR mTOR Ras Raf	Pri	France
Lou *et al* [31]	CSe	Pr	CC	531	PI3K Ras	M	Guatemala Venezuela Mexico
Wang *et al* [32]	CS	RT	CC	60	PI3K	Pu	China
Tornesello *et al* [33]	CSe	Pr	CC, PL	83	PI3K	Pu	Italy
Li *et al* [34]	CS	Pr	CC	24	EGFR	M	China
Wright *et al* [35]	CSe	Pr	CC	80	EGFR PI3K Ras	M	USA
McIntyre *et al* [36]	CS	Pr	CC	82	PI3K	M	Canada
Janku *et al* [37]	CS	Pr	CC, EC, OC, BC	17	PI3K Ras Raf	M	USA
Farley *et al* [38]	RCT	RT	CC	69	EGFR	M	USA
Longatto-Filhio *et al* [39]	CSe	Pr	CC	30	EGFR	M	Brazil
Schrevel *et al* [40]	CS	Pr	CC	103	EGFR	M	Netherlands
Halle *et al* [41]	CS	OC	CC	178	EGFR	Pu	Norway
Lida *et al* [42]	CSe	Pr	CC	111	EGFR Ras Raf	M	Japan
Wegman *et al* [43]	CS	Pr	CC	107	Ras	M	Sweden
Giordano *et al* [44]	CS	OC	CC	110	EGFR	M	Italy
Bodner *et al* [45]	CS	Pr	CC	39	EGFR	M	Austria
Eijsink *et al* [46]	CS	Pr	CC	290	EGFR Akt	M	Netherlands
El Hamdani *et al* [47]	CSe	Pr	CC	53	EGFR	Pri	Morocco
Noordhuis *et al* [48]	CS	RT	CC	375	EGFR Akt	M	Netherlands
Perez-Regadera *et al* [49]	CS	Other	CC	170	EGRF	M	Spain
Cui *et al* [50]	CSe	Pr	CC	184	PI3K	M	Sweden
Shen *et al* [51]	CSe	Pr	CC	53	EGFR	M	China
Zhang *et al* [52]	CSe	Pr	CC	31	PI3K	M	China
Baltazar *et al* [53]	CS	Pr	CC	129	EGFR	Pu	Brazil
Bellone *et al* [54]	CSe	Pr	CC	14	EGFR	M	USA
Cerciello *et al* [55]	CS	RT	CC	38	EGFR	M	Egypt
Fuchs *et al* [56]	CSe	Pr	CC	78	EGFR	NI	Germany
Bertelsen *et al* [57]	CSe	Other	CC, PL	31	PI3K Akt	NI	Norway
Mammas *et al* [13]	CSe	Pr	CC	9	Ras	M	Greece
Kim *et al* [58]	CS	Pr	CC	68	EGFR	M	Korea
Cho *et al* [59]	CSe	Pr	CC	84	EGFR	M	Korea
Ray *et al* [60]	CSe	Pr	CC, BC	50	EGFR	NI	India
Kim *et al* [61]	CS	Pr	CC	73	EGFR	M	Korea
Leung [62]	CS	Pr	CC	78	EGFR Ras	M	Hong Kong
Alonio *et al* [63]	CSe	Pr	CC	30	Ras	M	Argentina, Mexico
Ma *et al* [64]	CSe	Pr	CC	18	PI3K	M	Taiwan
Kim *et al* [65]	CS	Pr	CC	32	EGFR	M	Korea
Hove *et al* [66]	CSe	Pr	CC	22	EGFR	Pri	USA
Skomedal *et al* [67]	CSe	Pr	CC	74	EGFR	M	Norway
Kersemaekers *et al* [68]	CS	Pr	CC	136	EGFR	Pri	Netherlands
Biesterfeld *et al* [69]	CSe	Pr	CC	30	EGFR	M	Germany
Parker *et al* [70]	CSe	Pr	CC	32	Ras	Pu	USA
Kristensen *et al* [71]	CS	OC	CC	132	EGFR	Pri	Norway
Lee *et al* [72]	CS	Pr	CC	27	Ras	M	Korea
Tenti *et al* [73]	CSe	Pr	CC	67	Ras	Pu	Italy
Hale *et al* [74]	CS	Pr	CC	62	EGFR	M	England
Willis *et al* [75]	CSe	CS	CC	15	Ras	M	England
Koulos *et al* [76]	CS	Pr	CC	32	Ras	M	USA
Hayashi *et al* [77]	CSe	Pr	CC	52	EGFR Ras	NI	Japan
Sato *et al* [78]	CSe	Pr	CC, EC	7	EGFR	M	Japan
De La Rochefordiere *et al* [79]	RCT	OC	CC	54	PI3K Ras Raf	M	France
Yamashita *et al* [80]	CS	Pr	CC	57	EGFR	M	Japan
Oka *et al* [81]	CS	OC	CC	216	EGFR	Pu	Japan
Wistuba *et al* [82]	CSe	Pr	CC, PL	38	EGFR	M	Chile
Bauknecht *et al* [83]	CSe	Pr	CC, OC, EC, BC	40	EGFR	Pu	Germany
Wistuba and Capurro [84]	CSe	Pr	CC, PL	16	Ras	M	Chile
Razia *et al* [15]	CS	Pr	CC	124	Pi3K Ras Raf	M	Japan
Lachkar *et al* [85]	CS	OC	CC	59	Pi3K	M	Japan
Jiang *et al* [86]	CS	OC	CC	854	Ras	M	China
Spaans *et al* [87]	CSe	Pr	CC	137	Pi3K Ras	M	Indonesia
de Almeida *et al* [88]	CSe	Other	CC	10	EGFR	M	Brazil
Hodgson *et al* [89]	CS	Pr	CC	20	Pi3K Ras	M	Canada
Wei *et al* [90]	CSe	Pr	CC	60	EGFR	M	China
Zou *et al* [91]	CSe	Pr	CC	260	Ras	M	China
Ueda *et al* [92]	CS	OC	CC	43	EGFR	M	Japan

Abbreviations:

Study design: CS, Cohort study; CSe, Cross sessional; RCT, Randomised clinical trial.

Study objective: Pr, Prevalence; RT, Response to treatment; OC, Oncology result.

Inclusion criteria: CC, Cervical cancer; EC, Endometrial cancer; BC, Breast cancer; OC, Ovarian cancer; PL, Precursor lesions.

Financial source: Pri, Private; Pu, Public; M, Mixed; NI, No information available

**Figure 1.**
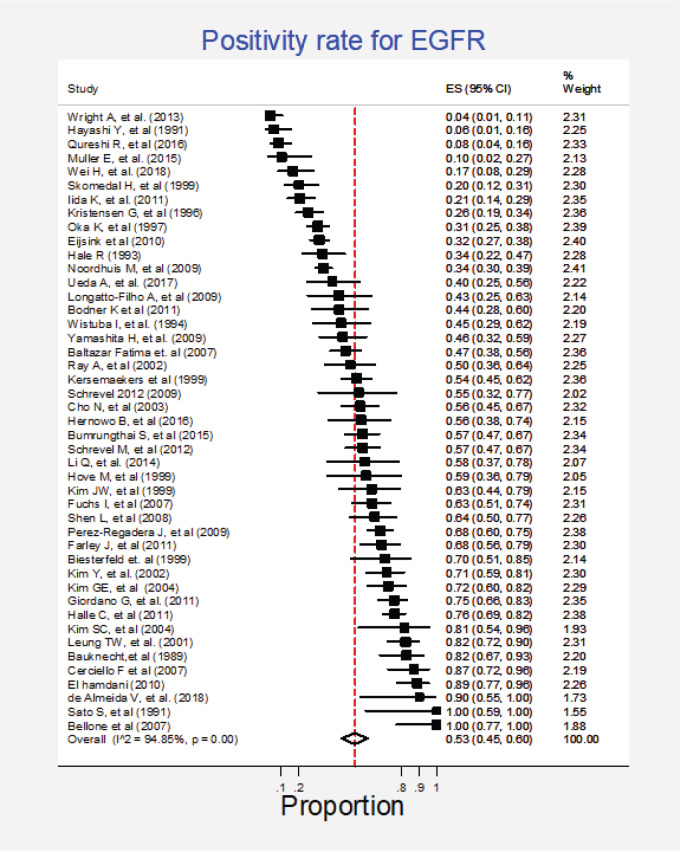
Meta-analysis of the EGFR positivity rates.

**Figure 2.**
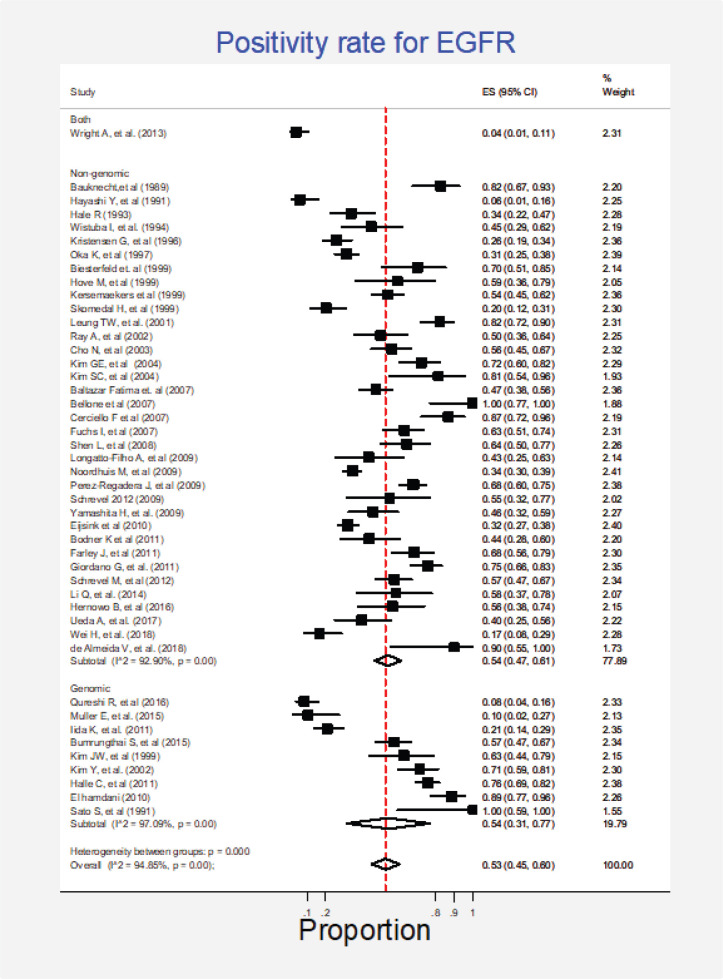
Subgroup analysis. Positivity for EGFR according to the type of detection test (genomic versus non-genomic).

## Appendix A. Search strategy.

Database: Ovid MEDLINE(R), Epub Ahead of Print, In-Process and Other Non-Indexed Citations, Ovid MEDLINE(R) Daily and Ovid MEDLINE(R) <1946 to Present>

Search strategy:

-------------------------------------------------------------------------------------------------------------------------------------------------------------------

1. exp Uterine Cervical Neoplasms/
2. (cervi* adj5 (cancer* or tumo?r* or neoplas* or carcinoma* or malignan* or adenocarcinoma* or choriocarcinoma* or teratoma* or sarcoma*)).ti,ab.
3. 1 or 2
4. exp Phosphatidylinositol 3-Kinases/
5. (Phosphatidylinositol 3 adj1 kinas*).ti,ab.
6. (PI3K* or PI 3K* or PI-3K* or PI 3-kinase* or PI3 kinase*).ti,ab.
7. (ptdlns adj3 kinase*).ti,ab.
8. phosphatidylinositol-3-oh kinase*.ti,ab.
9. 4 or 5 or 6 or 7 or 8
10. exp Genes, ras/
11. ras.ti,ab.
12. 10 or 11
13. exp Proto-Oncogene Proteins B-raf/
14. raf.ti,ab.
15. 13 or 14
16. kras protein, human.mp.
17. kras.ti,ab.
18. 16 or 17
19. exp Receptor Protein-Tyrosine Kinases/
20. (receptor* adj3 kinase*).ti,ab.
21. 19 or 20
22. exp Receptor, Epidermal Growth Factor/
23. (epiderm* adj3 factor*).ti,ab.
24. 22 or 23
25. exp ErbB Receptors/
26. (erbb adj3 receptor*).ti,ab.
27. 25 or 26
28. exp Genetic Variation/
29. (genet* adj3 (mutat* or variat*)).ti,ab.
30. (therap* adj3 target*).ti,ab.
31. 28 or 29 or 30
32. 9 or 12 or 15 or 18 or 21 or 24 or 27 or 31
33. 3 and 32
34. 9 or 12 or 15 or 18 or 21 or 24 or 27
35. 3 and 34

***************************

## Appendix B. Excluded studies.

The following table gathers the references of the studies that were excluded (*N* = 93) at the full text review stage. They are grouped according to reason for exclusion.

**Table:**

**Language other than English or Spanish (*N* = 8, 8.60%)**	
Chinese (*N* = 5)	Feng S, Zhang Y, et al. Expression of epidermal growth factor receptor and the correlation with HPV16/18 infection in cervical intraepithelial neoplasia and cervical carcinoma. Zhonghua Zhong Liu Za Zhi. 2007 Oct;29(10):759-63.
	Yao T, Dai Y, et al. Expression and clinical significance of phosphatidylinositol 3-kinase and protein kinase B in cervical carcinoma. Ai Zheng. 2008 May;27(5):525-30.
	Zhou S, Feng G, et al. Analysis of epidermal growth factor receptor expression and gene expression status in tissue microarray of cervical squamous cell carcinoma. Zhonghua Fu Chan Ke Za Zhi. 2013 Nov;48(11):843-6.
	Xiao-hong M, Xiao-yan Z, et al. Expressions of phosphatidylinositol 3 kinase and phosphorylated Akt in condyloma acuminatum and cervical squamous cell carcinoma. Zhonghua Pifuke Zazhi 2011;44(12):857-860.
	Zhao J, Ma, Kuerban D, et al. Correlation of expressions of EGFR, c-jun, and c-fos of cervical squamous cell carcinomas and clinical features and short-term efficacy. J. Shanghai Jiaotong Univ. Med. Sci. 2015;35(2):216-222 and 232.
Japanese (*N* = 1)	Sugimura M, Kobayashi H, et al. Immunohistochemical study on the expression of epidermal growth factor receptor (EGF-R) in invasive cervical cancer of the uterus. Nihon Sanka Fujinka Gakkai Zasshi. 1992 Jun;44(6):689-94.
German (*N* = 1)	Stellwag B, Scheidel P, et al. EGF receptor and EGF-like activity as prognostic factors in cervix cancer. Geburtshilfe Frauenheilkd. 1993 Mar;53(3):177-81.
Portuguese (*N* = 1)	Fukazawa E. Avaliação do controle local dos carcinomas de células escamosas avançados do colo do útero submetido à radioterapia através da análise imunoistoquimica da COX-2, EGFR, CD44v6, HIF-1 e HIF2 São Paulo; 2012, (1):90-90.
**Unavailable full text** (*N* = 7, 7.52%)	
Altavilla C, Castellan L, et al. Prognostic significance of epidermal growth factor receptor (EGFR) and c-erbB-2 protein overexpression in adenocarcinoma of the uterine cervix. Eur J Gynaecol Oncol. 1996;17(4):267-70.	
Melczer Z, F Bánhidy, et al. ErbB-2/HER-2 protein expression, serum tumour necrosis factor-alpha (TFM-alpha) and soluble tumour necrosis factor receptor-2 (TNFR-2) concentrations in human carcinoma of the uterine cervix. Eur J Gynaecol Oncol. 2003;24(2):138-42.	
Mulhall C, Coward J, et al. Targeted therapeutic management of locally advanced, recurrent and metastatic cervical cancer. Cerv. Cancer: Screen. Methods, Risk Factors and Treat. Options 2013:199-226. Nova Science Publishers, Inc. 2013.	
Riou G, Sheng Z, et al. c-myc and c-Ha-ras proto-oncogenes in invasive cervical cancers: Prognostic significance. Bull. Cancer;77(4):341-347.	
Wistuba I, Roa I, et al. Immunohistochemical expression of epidermal growth factor receptor (EGFR) in epidermoid carcinoma of the cervix uteri and its precursor lesions. Rev Chil Obstet Ginecol. 1994;59(2):116-22.	
Yamashita H, Murakami N, et al. Correlation among six biologic factors (p53, p21(WAF1), MIB-1, EGFR, HER2, and Bcl-2) and clinical outcomes after curative chemoradiation therapy in squamous cell cervical cancer. Int J Radiat Oncol Biol Phys.2009 Jul 15;74(4):1165-72.	
Duan B, Zhang Y, et al. Clinical significance of next-generation sequencing for advanced cancer. Chin. J. Cancer Prev. Treat. 2017;24(7):458-463. Chinese Journal of Cancer Prevention and Treatment, Editorial board 2017.	
**Unextractable data** (*N* = 17, 18.27%)	
Nogueira-Rodrigues A, Moralez G, et al. Phase 2 trial of erlotinib combined with cisplatin and radiotherapy in patients with locally advanced cervical cancer. Cancer. 2014 Apr 15;120(8):1187-93.	
Juric D, Rodon J, et al. Phosphatidylinositol 3-Kinase α-Selective Inhibition With Alpelisib (BYL719) in PIK3CA-Altered Solid Tumors: Results From the First-in-Human Study. J Clin Oncol.2018 May 1;36(13):1291-1299.	
Lu H, Wu Y, et al. A prospective study on neoadjuvant chemoradiotherapy plus anti-EGFR monoclonal antibody followed by surgery for locally advanced cervical cancer. Onco Targets Ther.2018 Jul 2;11:3785-3792.	
Meyer H, Gundermann P, et al. Associations between whole tumor histogram analysis parameters derived from ADC maps and expression of EGFR, VEGF, Hif 1-alpha, Her-2 and Histone 3 in uterine cervical cancer. Magn Reson Imaging. 2019 Apr;57:68-74.	
Meyer H, Hamerla G, et al. Whole Lesion Histogram Analysis Derived From Morphological MRI Sequences Might be Able to Predict EGFR- and Her2-Expression in Cervical Cancer. Acad Radiol. 2019 Aug;26(8):e208-e215.	
Nuryadi E, Sasaki Y, et al. Mutational analysis of uterine cervical cancer that survived multiple rounds of radiotherapy. Oncotarget. 2018 Aug 24;9(66):32642-32652.	
Pignata S, Scambia G, et al. The MITO CERV-2 trial: A randomized phase II study of cetuximab plus carboplatin and paclitaxel, in advanced or recurrent cervical cancer. Gynecol Oncol. 2019 Jun;153(3):535-540.	
Pillai M, Jayaprakash P, et al. Tumour-proliferative fraction and growth factor expression as markers of tumour response to radiotherapy in cancer of the uterine cervix. J Cancer Res Clin Oncol. 1998;124(8):456-61.	
Surov A, Meyer J, et al. Metabolo-volumetric parameters of 18F-FDG-PET can predict expression of EGFR and HIF 1alpha in uterine cervical cancer. Cancer Biomark. 2019;24(1):135-140.	
Grigsby P, Elhammali A, et al. Clinical outcomes and differential effects of PI3K pathway mutation in obese versus non-obese patients with cervical cancer. Oncotarget. 2017 Dec 23;9(3):4061-4073.	
Tinker A, Ellard S, et al. Phase II study of temsirolimus (CCI-779) in women with recurrent, unresectable, locally advanced or metastatic carcinoma of the cervix. A trial of the NCIC Clinical Trials Group (NCIC CTG IND 199). Gynecol Oncol. 2013 Aug;130(2):269-74.	
Gaffney D, Haslam D, et al. Epidermal growth factor receptor (EGFR) and vascular endothelial growth factor (VEGF) negatively affect overall survival in carcinoma of the cervix treated with radiotherapy. Int J Radiat Oncol Biol Phys. 2003 Jul 15;56(4):922-8.	
Genetic alterations and PIK3CA gene mutations and amplifications analysis in cervical cancer by racial groups in the United States. Florence Femi O. Int J Health Sci. Jan-Feb 2018;12(1):28-32.	
Jiang W, He T, et al. The PIK3CA E542K and E545K mutations promote glycolysis and proliferation via induction of the β-catenin/SIRT3 signaling pathway in cervical cancer. J Hematol Oncol. 2018 Dec 14;11(1):139.	
Nagy V, Buiga R, et al. Expression of VEGF, VEGFR, EGFR, COX-2 and MVD in cervical carcinoma, in relation with the response to radio-chemotherapy. Rom J Morphol Embryol. 2011;52(1):53-9.	
Ojesina A, Lichtenstein L, et al. Landscape of genomic alterations in cervical carcinomas. Nature. 2014 Feb 20;506(7488):371-5.	
Soh L, Heng D, et al. The relevance of oncogenes as prognostic markers in cervical cancer. Int J Gynecol Cancer. Sep-Oct 2002;12(5):465-74.	
**Inappropriate study population for research question** (*N* = 44, 47.31%)	
Experimental cancer cell cultures (e.g. HeLa), animal cell lines (N = 4)	Adefuye A, Sales K, et al. Seminal plasma induces the expression of IL-1α in normal and neoplastic cervical cells via EP2/EGFR/PI3K/AKT pathway. J Mol Signal. 2014 Aug 8;9:8.
	Arias-Pulido H, Joste N, et al. Absence of epidermal growth factor receptor mutations in cervical cancer. Int J Gynecol Cancer.Jul-Aug 2008;18(4):749-54.
	Arjumand W, Merry C, et al. Phosphatidyl inositol-3 kinase (PIK3CA) E545K mutation confers cisplatin resistance and a migratory phenotype in cervical cancer cells. Oncotarget. 2016 Dec 13;7(50):82424-82439.
	Meira D, de Almeida V, et al. Combination of cetuximab with chemoradiation, trastuzumab or MAPK inhibitors: mechanisms of sensitisation of cervical cancer cells. Br J Cancer. 2009 Sep 1;101(5):782-91.
Incorrect intracellular pathway, crosstalk between pathways or HPV expression only (*N* = 32)	Araújo A, Raquel Catarino R, et al .Epidermal growth factor genetic variation associated with advanced cervical cancer in younger women. Am J Clin Oncol. 2012 Jun;35(3):247-50.
	Banister C, Changlong Liu C, et al. Identification and characterization of HPV-independent cervical cancers. Oncotarget. 2017 Feb 21;8(8):13375-13386.
	Hale R, Buckley C, et al. Prognostic value of c-erbB-2 expression in uterine cervical carcinoma. J Clin Pathol.1992 Jul;45(7).
	Kihana T, Tsuda H, et al. Prognostic significance of the overexpression of c-erbB-2 protein in adenocarcinoma of the uterine cervix. Cancer.1994 Jan 1;73(1):148-53.
	Lee C, Lee J, et al. Expression of HER2neu (c-erbB-2) and epidermal growth factor receptor in cervical cancer: prognostic correlation with clinical characteristics, and comparison of manual and automated imaging analysis. Gynecol Oncol. 2004 Apr;93(1):209-14.
	Lee C, Shrieve D, et al. Correlation between human epidermal growth factor receptor family (EGFR, HER2, HER3, HER4), phosphorylated Akt (P-Akt), and clinical outcomes after radiation therapy in carcinoma of the cervix. Gynecol Oncol. 2005 Nov;99(2):415-21.
	Liu D, Li F, et al. Increased RIPK4 expression is associated with progression and poor prognosis in cervical squamous cell carcinoma patients. Sci Rep. 2015 Jul 7;5:11955.
	Mammas I, Zafiropoulos A, et al. Human papillomavirus (HPV) typing in relation to ras oncogene mRNA expression in HPV-associated human squamous cervical neoplasia. Int J Biol Markers. Oct-Dec 2005;20(4):257-63.
	Niibe Y, Watanabe J, et al. Concomitant expression of HER2 and HIF-1alpha is a predictor of poor prognosis in uterine cervical carcinoma treated with concurrent chemoradiotherapy: prospective analysis (KGROG0501). Eur J Gynaecol Oncol. 2010;31(5):491-6.
	Nishioka T, West C, et al. Prognostic significance of c-erbB-2 protein expression in carcinoma of the cervix treated with radiotherapy. J Cancer Res Clin Oncol. 1999;125(2):96-100.
	Pérez-Regadera J, Sánchez-Muñoz A, et al. Cisplatin-based radiochemotherapy improves the negative prognosis of c-erbB-2 overexpressing advanced cervical cancer. Int J Gynecol Cancer. 2010 Jan;20(1):164-72.
	Hellberg D, Tot T. Tumor marker score for prognostication of early-stage squamous cell cervical cancer. Anticancer Res.2014 Feb;34(2):887-92.
	Ngan H, Cheung A, et al. Abnormal expression of epidermal growth factor receptor and c-erbB2 in squamous cell carcinoma of the cervix: correlation with human papillomavirus and prognosis. Tumour Biol. May-Jun 2001;22(3):176-83.
	Aoyama C, Peters J, et al. Uterine cervical dysplasia and cancer: identification of c-myc status by quantitative polymerase chain reaction. Diagn Mol Pathol. 1998 Dec;7(6):324-30.
	Bellone S, Palmieri M, et al. Selection of HER-2/neu-positive tumor cells in early stage cervical cancer: implications for Herceptin-mediated therapy. Gynecol Oncol. 2003 Oct;91(1):231-40.
	Berchuck A, Rodriguez G, et al. Expression of epidermal growth factor receptor and HER-2/neu in normal and neoplastic cervix, vulva, and vagina. Obstet Gynecol. 1990 Sep;76(3 Pt 1):381-7.
	Bhadauria M, Ray A, et al. Oncoprotein c-erbB-2 in squamous cell carcinoma of the uterine cervix and evaluation of its significance in response of disease to treatment. Indian J Physiol Pharmacol. 2001 Apr;45(2):191-8.
	Braicu E, Fotopoulou C, et al. Role of serum concentration of VEGFR1 and TIMP2 on clinical outcome in primary cervical cancer: results of a companion protocol of the randomized, NOGGO-AGO phase III adjuvant trial of simultaneous cisplatin-based radiochemotherapy vs. carboplatin and paclitaxel containing sequential radiotherapy. Cytokine.2013 Mar;61(3):755-8.
	Branca M, Ciotti M, et al. Predicting high-risk human papillomavirus infection, progression of cervical intraepithelial neoplasia, and prognosis of cervical cancer with a panel of 13 biomarkers tested in multivariate modeling. Int J Gynecol Pathol. 2008 Apr;27(2):265-73.
	Califano D, Losito S, et al. Significance of erb-B2 immunoreactivity in cervical cancer. Front Biosci.2006 Sep 1;11:2071-6.
	Cancer Genome Atlas Research Network. Integrated genomic and molecular characterization of cervical cancer. Nature. 2017 Mar 16;543(7645):378-384. Chen J. Signaling pathways in HPV-associated cancers and therapeutic implications. Rev Med Virol.2015 Mar;25 Suppl 1:24-53.
	Guo L, Wu H, et al. Genetic variations in the PI3K/AKT pathway predict platinum-based neoadjuvant chemotherapeutic sensitivity in squamous cervical cancer. Life Sci.2015 Dec 15;143:217-24.
	Mathur S, Mathur R, et al. Human papilloma virus (HPV)-E6/E7 and epidermal growth factor receptor (EGF-R) protein levels in cervical cancer and cervical intraepithelial neoplasia (CIN). Am J Reprod Immunol. 2001 Oct;46(4):280-7.
	O'Leary J, Landers R, et al. Molecular analysis of ras oncogenes in CIN III and in stage I and II invasive squamous cell carcinoma of the uterine cervix. J Clin Pathol. 1998 Aug;51(8):576-82.
	Pochylski T, Kwaśniewska A. Absence of point mutation in codons 12 and 13 of K-RAS oncogene in HPV-associated high grade dysplasia and squamous cell cervical carcinoma. Eur J Obstet Gynecol Reprod Biol. 2003 Nov 10;111(1):68-73.
	Sato S, Ito K, et al. Analysis of point mutations at codon 12 of K-ras in human endometrial carcinoma and cervical adenocarcinoma by dot blot hybridization and polymerase chain reaction. Tohoku J Exp Med. 1991 Oct;165(2):131-6.
	Schwarz J, Payton J, et al. Pathway-specific analysis of gene expression data identifies the PI3K/Akt pathway as a novel therapeutic target in cervical cancer. Clin Cancer Res.2012 Mar 1;18(5):1464-71.
	Shu X, Wu J, et al. PAK4 confers the malignance of cervical cancers and contributes to the cisplatin-resistance in cervical cancer cells via PI3K/AKT pathway. Diagn Pathol.2015 Sep 28;10:177.
	Ueno S, Sudo T, et al. Absence of human papillomavirus infection and activation of PI3K-AKT pathway in cervical clear cell carcinoma. Int J Gynecol Cancer. 2013 Jul;23(6):1084-91.
	Vosmik M, Laco J, et al. Prognostic significance of human papillomavirus (HPV) status and expression of selected markers (HER2/neu, EGFR, VEGF, CD34, p63, p53 and Ki67/MIB-1) on outcome after (chemo-) radiotherapy in patients with squamous cell carcinoma of uterine cervix. Pathol Oncol Res.2014 Jan;20(1):131-7.
	Wong Y, Chung T, et al. Frequent ras gene mutations in squamous cell cervical cancer. Cancer Lett.1995 Aug 16;95(1-2):29-32.
	An J, Man-Ni Huang M, et al. A preliminary study of genes related to concomitant chemoradiotherapy resistance in advanced uterine cervical squamous cell carcinoma. Chin Med J (Engl).2013 Nov;126(21):4109-15.
Preinvasive cervical lesions or cytology (*N* = 5)	Balan R, Simion N, et al. Immunohistochemical assessment of p16, COX-2 and EGFR in HPV-positive cervical squamous intraepithelial lesions. Rom J Morphol Embryol. 2011;52(4):1187-94.
	Dokianakis D, Papaefthimiou M, et al. High prevalence of HPV18 in correlation with ras gene mutations and clinicopathological parameters in cervical cancer studied from stained cytological smears. Oncol Rep. Nov-Dec 1999;6(6):1327-31.
	García D, Arias Y, et al. Detection of genes mutations in the K-ras, H-ras and EGFR in samples of blood plasma and cervical smears for patients with cervical intraepithelial neoplasia III and cervical cancer. Colomb. Med. 2009;40(1):34-50.
	García D, Arias Y, et al. Detección de mutaciones en los genes K-ras, H-ras y EGFR en muestras de plasma sanguíneo y cepillado cervical de pacientes con neoplasia intraepitelial cervical (NIC) III y cáncer de cuello uterino. Colomb. Med. vol.40 no.1 Cali Jan./Mar. 2009.
	García D, Briceño I, et al. Detection of gene amplification in MYCN, C-MYC, MYCL1, ERBB2, EGFR, AKT2, and human papilloma virus in samples from cervical smear normal cytology, intraepithelial cervical neoplasia (CIN I, II, III), and cervical cancer. Colomb Med (Cali) 2011;42(2):144-153.
Recurrent cervical cancer (*N* = 3)	Hasegawa K, Kagabu M, et al. Phase II basket trial of perifosine monotherapy for recurrent gynecologic cancer with or without PIK3CA mutations. Invest New Drugs.2017 Dec;35(6):800-812.
	Liu J, Gray K, et al. Results from a single arm, single stage phase II trial of trametinib and GSK2141795 in persistent or recurrent cervical cancer. Gynecol Oncol. 2019 Jul;154(1):95-101.
	Goncalves A, Fabbro M, et al. A phase II trial to evaluate gefitinib as second- or third-line treatment in patients with recurring locoregionally advanced or metastatic cervical cancer. Gynecol Oncol. 2008 Jan;108(1):42-6.
**Study design inappropriate for the research question** (*N* = 17, 18.27%)	
Narrative reviews (*N* = 11)	Bahrami A, Hasanzadeh M, et al. The Potential Value of the PI3K/Akt/mTOR Signaling Pathway for Assessing Prognosis in Cervical Cancer and as a Target for Therapy. J Cell Biochem. 2017 Dec;118(12):4163-4169.
	Barra F, Lorusso D, et al. Investigational drugs for the treatment of cervical cancer. Expert Opin Investig Drugs. 2017 Apr;26(4):389-402.
	Boussios S, Seraj E, et al. Management of patients with recurrent/advanced cervical cancer beyond first line platinum regimens: Where do we stand? A literature review. Crit Rev Oncol Hematol. 2016 Dec;108:164-174.
	Bregar A, Growdon W. Emerging strategies for targeting PI3K in gynecologic cancer. Gynecol Oncol. 2016 Feb;140(2):333-44.
	Husseinzadeh N, Husseinzadeh D. mTOR inhibitors and their clinical application in cervical, endometrial and ovarian cancers: a critical review. Gynecol Oncol.2014 May;133(2):375-81.
	Mammas I, Zafiropoulos A, et al. Involvement of the ras genes in female genital tract cancer (Review). Int J Oncol. 2005 May;26(5):1241-55.
	Manzo-Merino J, Contreras-Paredes A, et al. The role of signaling pathways in cervical cancer and molecular therapeutic targets. Arch Med Res. 2014 Oct;45(7):525-39.
	Satoshi Takeuchi. Biology and treatment of cervical adenocarcinoma. Chin J Cancer Res. 2016 Apr;28(2):254-62.
	Salvesen H, Werner H, et al. PI3K pathway in gynecologic malignancies. Am Soc Clin Oncol Educ Book.2013.
	Soonthornthum T, Arias-Pulido H, et al. Epidermal growth factor receptor as a biomarker for cervical cancer. Ann Oncol. 2011 Oct;22(10):2166-78.
	Wu J, Chen C, et al. Phosphatidylinositol 3-kinase signaling as a therapeutic target for cervical cancer. Curr Cancer Drug Targets.2013 Feb;13(2):143-56.
Systematic reviews (*N* = 4)	Zagouri F, Sergentanis T, et al. Molecularly targeted therapies in cervical cancer. A systematic review. Gynecol Oncol. 2012 Aug;126(2):291-303.
	Zheng X, Du X. Prognostic significance of EGFR in cervical cancer: a Meta-analysis. Chin. J. Cancer Prev. Treat. 2015;22(16):1321-1327 and 1334.
	Noordhuis M, Eijsink J, et al. Prognostic cell biological markers in cervical cancer patients primarily treated with (chemo)radiation: a systematic review. Int J Radiat Oncol Biol Phys. 2011 Feb 1;79(2):325-34.
	Tian W, Huang M, et al. Prognostic Impact of Epidermal Growth Factor Receptor Overexpression in Patients with Cervical Cancer: A Meta-Analysis. PLoS One.2016 Jul 20;11(7):e0158787.
Letters to the editor (*N* = 2)	Al Moustafa A. Human cervical carcinomas and EGF-R tyrosine kinase inhibitors. Gynecologic oncology / 2008;109(2):308.
	Van de Putte G, Vach W. P63 and EGFR as prognostic predictors in stage IIB radiation-treated cervical squamous cell carcinoma. Gynecol Oncol. 2005 Aug;98(2):335-6.

## Appendix C. Study methodology evaluation tool used in this review.

**Table:**

Item	Yes	No	Unclear	Not applicable
1	Was the sample frame appropriate to address the target population? Description of the specific characteristics of the target study population that are relevant to the main subject: patients diagnosed with uterine cervical cancer. Yes No *Assesses selection bias			
2	Were study participants recruited in an appropriate way? Description of the data source, baseline data of the tumour sources, for example, obtained from a tumour bank or pathology laboratory. Yes No = Uninformed *Assesses selection bias			
3	Was the sample size adequate? Description of the sample size calculation, if appropriate for the primary study subject. Yes (sample size adequately calculated) No (sample size inadequately calculated) Unclear (not expressed) Not applicable (irrelevant for the primary study subject) *Assesses precision			
4	Were the study subjects and setting described in detail? Description of the study sample with sufficient detail so that other researchers may determine whether the population is relevant to them. E.g.: age at diagnosis, tumour state, tumour histology. Yes No (insufficiently described) Unclear (no description) *Assesses reporting quality			
5	Was data analysis conducted with sufficient coverage of the identified sample? Considering the amount of unevaluated or unincluded samples, could the loss lead to a sub estimation of the mutation prevalence? Yes: sample loss is less than 10% No: sample loss is greater than 10% *Assesses data loss bias.			
6	Was the condition measured in a standard, reliable way for the variable of interest in all cases? Description of the methods used for the identification of mutations. Were standard methods used? E.g. immunohistochemistry, fluorescence in-situ hybridisation, PCR, commercial probes, etc. Yes Unclear *Assesses information bias.			
7	Was there appropriate statistical analysis? Description of the statistical methods and analysis tools used, relevant to the study subject. Yes (performed appropriately) No (performed incorrectly) Unclear *Assesses internal validity			

## Appendix D. Figures.

**EGFR**

**PI3K**

**Ras**

**Akt**

**Raf**
